# The underground tango: How ethylene and auxin interact to regulate cereal root angle

**DOI:** 10.1093/plphys/kiae194

**Published:** 2024-04-01

**Authors:** Aida Maric

**Affiliations:** Assistant Features Editor, Plant Physiology, American Society of Plant Biologists; CIBSS—Centre for Integrative Biological Signalling Studies, University of Freiburg, 79104, Freiburg, Germany; Plant Environmental Signalling and Development, Institute of Biology III, University of Freiburg, 79104 Freiburg, Germany

Roots are the underground navigation system of the plant. From flooding-prone regions to drought-susceptible areas and nutrient-poor soils, root architecture drives acclimation and stress mitigation. Roots optimize water acquisition in drought conditions by changing root growth angle and directing roots toward deeper soil layers, avoiding drought in upper soil layers and accessing water in deeper soil. Flooded rice plants adjust the root angle and grow away from darkness, therefore avoiding deeper soil with lower oxygen concentrations; and they grow away from light, thereby mitigating desiccation upon water retrieval (Pedersen et al. 2020). These root angle adjustments direct the root growth to the area close to the soil surface.

Root responses to environmental stimuli are guided by the interplay of phytohormones. Ethylene and auxin play especially important roles in the regulation of root architecture. Ethylene is a gaseous hormone with a role in the regulation of stress responses and controls the plant response to stimuli such as drought or flooding. Auxin is a very well-known regulator of plant growth, among others regulating root gravitropism response. Moreover, the ethylene-mediated regulation of auxin biosynthesis has been reported before, but how they interact to regulate root angle is still unknown, especially in crop plants (Ruzicka et al. 2007; Stepanova et al. 2005).

Recent work by Kong et al. (2024) published in *Plant Physiology* reports ethylene-auxin hormonal crosstalk as a regulator of root angle in cereal crops. The authors used different root phenotyping methods to compare root response of wild-type and ethylene-insensitive rice and maize mutants. EIL1 and EIN2 are crucial links in the ethylene signaling pathway, conducting the ethylene signal from the endoplasmic reticulum to the nucleus to control ethylene-responsive genes. Rice and maize mutants of EIL1 and EIN2 (*oseil1*, *osein2*, and *zmein2-1*) were not able to sense ethylene signals and had impaired ethylene response. Kong et al. (2024) showed that *oseil1* and *osein2 mutants* have a shallower root system with larger root angles (Fig. A). Additionally, ethylene mutants had an impaired gravitropic response compared to wild-type plants. These root responses in cereals contrast the root phenotype in Arabidopsis (Buer et al. 2006). Unlike in rice and maize, ethylene signaling mutants of Arabidopsis showed no gravitropism defects both in the presence and absence of the ethylene precursor ACC. Surprisingly, another recent report showed that ethylene signalling does lead to more horizontal lateral root angles in Arabidopsis (He et al. 2024).

**Figure.**
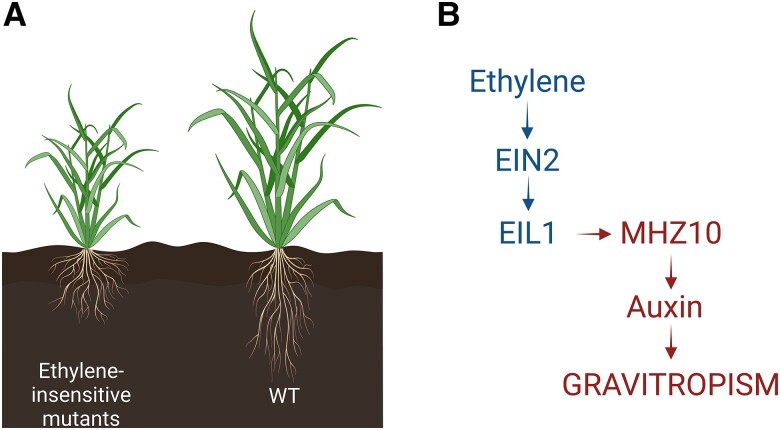
Ethylene mediated regulation of root angle by regulation of auxin. **A)** Ethylene-insensitive mutants have shallower root crown with larger root angles compared to wild type. **B)** Ethylene accumulation in plants leads to activation of ethylene signaling pathway through EIN2 and EIL1, which then activates MHZ10, an auxin biosynthesis gene. In turn, auxin regulates gravitropism response of the root system.

In an attempt to understand the dynamics of ethylene regulation of rice root angle, the authors performed hormonal profiling.By comparing hormone profiles of ethylene-insensitive mutants with wild-type plants they showed that ethylene-insensitive mutants exhibit lower levels of auxin. Exogenous application of auxin could rescue the root angle phenotype of the ethylene-insensitive mutants (Fig. B), pinpointing auxin as the intermediate signal in ethylene-mediated root growth angles. Furthermore the authors showed that *MHZ10*, a member of the auxin biosynthesis pathway that plays a key role in the ethylene-mediated regulation of root angle (Fig. B).

This study by Kong et al. (2024) uncovers MHZ10 as a regulator of hormonal crosstalk between ethylene and auxin to regulate root angle. This and similar studies could be the basis for the development of crops with improved root systems that can optimize the uptake of nutrients and have improved resilience in the face of the ongoing climate crisis. Moreover, the hormonal crosstalk regulating the root architecture described in this paper can be a basis for understanding the conserved and distinct mechanisms that control root angle between monocots and dicots. In the context of climate change, it is very appealing to envision a future with a wide range of crops such as potato and wheat being able to tune their root architecture to survive both too wet and dry soil conditions. Bearing in mind that other factors such as the microbiome (Gonin et al. 2023), oxygen status (Daniel and Hartman 2024), salt and nutrients (Buer et al. 2003) converge on ethylene and auxin to regulate root angle and general architecture, future studies will have the task of exploring their interaction and potential to regulate the resilience of plants and maximize nutrient and water uptake. Figure adapted from Kong et al. (2024). Created with BioRender.com.
